# Fabrication of Gelatin-Based Electrospun Composite Fibers for Anti-Bacterial Properties and Protein Adsorption

**DOI:** 10.3390/md14100192

**Published:** 2016-10-21

**Authors:** Ya Gao, Yingbo Wang, Yimin Wang, Wenguo Cui

**Affiliations:** 1 College of Chemical Engineering, Xinjiang Normal University, Urumqi 830054, China; gaoya1965810837@sina.com (Y.G.); wangyimin73@sina.com (Y.W.); 2 Department of Orthopedics, The First Affiliated Hospital of Soochow University, Orthopedic Institute, Soochow University, 708 Renmin Road, Suzhou 215006, China

**Keywords:** electrospinning, composite fibers, antibacterial properties, protein adsorption

## Abstract

A major goal of biomimetics is the development of chemical compositions and structures that simulate the extracellular matrix. In this study, gelatin-based electrospun composite fibrous membranes were prepared by electrospinning to generate bone scaffold materials. The gelatin-based multicomponent composite fibers were fabricated using co-electrospinning, and the composite fibers of chitosan (CS), gelatin (Gel), hydroxyapatite (HA), and graphene oxide (GO) were successfully fabricated for multi-function characteristics of biomimetic scaffolds. The effect of component concentration on composite fiber morphology, antibacterial properties, and protein adsorption were investigated. Composite fibers exhibited effective antibacterial activity against *Staphylococcus aureus* and *Escherichia coli*. The study observed that the composite fibers have higher adsorption capacities of bovine serum albumin (BSA) at pH 5.32–6.00 than at pH 3.90–4.50 or 7.35. The protein adsorption on the surface of the composite fiber increased as the initial BSA concentration increased. The surface of the composite reached adsorption equilibrium at 20 min. These results have specific applications for the development of bone scaffold materials, and broad implications in the field of tissue engineering.

## 1. Introduction

Human bones are composed of organic and inorganic components, particularly hydroxyapatite (HA) and collagen. Composite materials that mimic the bone matrix have important clinical applications. HA exhibits excellent biocompatibility and biodegradability and, therefore, is a high-profile artificial bone material. However, its insufficient flexural and compressive strength and high brittleness limit its medical applications [1]. Gelatin (Gel) is a modified collagen product and a natural polymer; it is structurally similar to collagen in extracellular matrices [2,3,4]. These composite materials can adapt well to the internal environment of humans [5]. Gel is rich in amino and carboxyl hydrophilic groups [6] and is beneficial for nutrients and oxygen infiltration. Chitosan (CS) possesses good biocompatibility, antimicrobial properties [7], and has the potential for various chemical modifications and combinations to obtain specific properties [8]. Accordingly, it has a wide range of applications in tissue engineering [9].

Graphene oxide (GO) is a derivative of graphene. Carboxylic, epoxy, and hydroxyl groups, in addition to many other highly active response groups, facilitate the combination of GO with other substances to form new composite materials. GO is widely used for biomedical applications. Owing to its high-strength mechanical properties, GO can be used in medical implants, as a filler, or as reinforcement material in tissue engineering scaffolds [10]. GO also has excellent antibacterial properties [11] and can be used for external wound healing to prevent infection [12]. GO-based composite nanofibers have been successfully prepared by electrospinning. Lu et al. [13] fabricated reduced graphene oxide (RGO)/CS/polyvinyl alcohol nanofiber scaffolds for wound healing and observed that RGO is beneficial for cellular attachment and growth. Andreia et al. [14] developed a nanocomposite comprised of GO sheets with silver nanoparticles (GO-Ag), and found that it can inhibit the growth of microbial adherent cells, thus preventing biofilm formation; however, the sudden release of silver was observed. Isis et al. [15] prepared GO/polyvinyl carbazole nanocomposites using electrochemical technology, and these had stronger antimicrobial effects than unmodified GO. According to these previous results, GO and RGO are beneficial for cell adhesion and growth. GO possesses antibacterial activity and can combine with polymers to form composites with favorable antimicrobial properties.

RGO and GO interact with the phospholipid bilayer of cells to form a stable structure [16], and the large specific surface area can promote cell adsorption [17], enhance cell adhesion, and induce proliferation via extracellular matrix protein adsorption [18]. In addition, the non-biodegradable RGO and GO can be discharged through lysosomes and enter the cell via phagocytes [19].

**Scheme 1:**
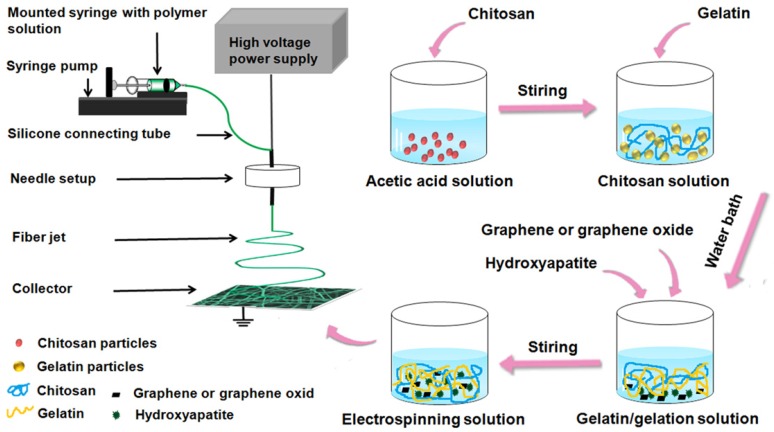
Schematic illustration of the fabrication process of Gel/CS/HA/GO composite fibers.

In this study, we demonstrate the design and facile fabrication of Gel/CS/HA/GO and Gel/CS/HA/RGO composite fibers using co-electrospinning, as shown in Scheme 1. With the development of materials science, biological materials have progressed from those that are passively adapted to the biological environment to those that are purposefully designed, with respect to material composition and microstructure, to confer specific functions [20]. Fibers generated by electrospinning are similar to the extracellular matrix with respect to structural morphology. The multipath structure in the Scheme 1 is similar to the collagen fiber structure of the extracellular matrix, which can be used for cellular attachment and growth [21]. At the same time, the large specific surface-area of the nanofibrous scaffold enhances its protein absorption ability, which is vital for cell anchoring [22]. Inorganic HA in bone tissue promotes bone remodeling via cell signaling to regulate osteoblast formation. This process is associated with an extracellular matrix protein adsorption effect. HA adsorption on these specific proteins can promote osteoblast proliferation, differentiation, and adhesion [23]. Accordingly, the adsorption of proteins has a very important effect on osteoblasts; it is, therefore, necessary to prepare a composite fiber with a similar composition to that of bone tissue that has good antibacterial properties and protein adsorption performance for use as a bone scaffold material. In this study, for the first time, the advantages of four materials: HA, Gel, CS, and GO, were combined to prepare composite nanofibers using electrospinning technology. The antimicrobial properties and the protein adsorption performance of these nanofibers were evaluated.

## 2. Results

### 2.1. Influence of Gel Concentration on Fibers

**Figure 1:**
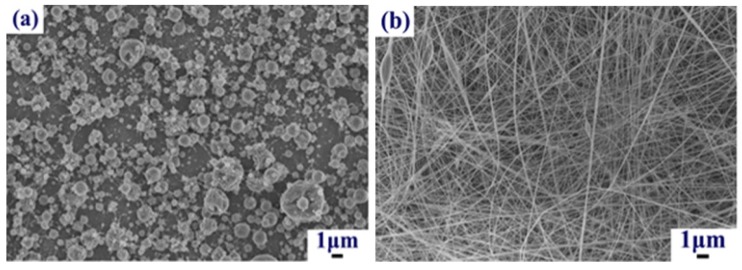

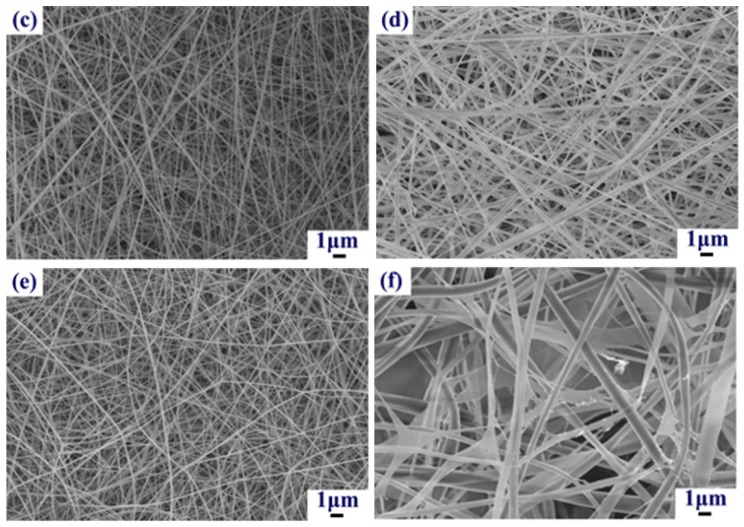
Scanning electron microscope (SEM) micrographs of fibers prepared with a series of Gel concentration; (**a**) 5 wt. %; (**b**) 10 wt. %; (**c**) 15 wt. %; (**d**) 20 wt. %; (**e**) 25 wt. %; (**f**) 30 wt. %.

Gel was the main component in the composite fiber in this experiment. The Gel concentration was the main factor determining the morphology of the composite fiber. Figure 1 shows that low Gel concentrations (i.e., ≤5 wt. %) resulted in insufficient surface tension, and the phenomenon of “electrospray” was observed (Figure 1a). As the Gel concentration increased (10 wt. %), surface tension gradually increased, and fiber formation was observed. There were some fiber junctions and bead structures in the fibers (Figure 1b). When the Gel concentration reached 15–20 wt. %, uniform fibers were obtained (Figure 1c,d). However, further increases in the Gel concentration (25 wt. %) led to increased surface tension. The electric force could not overcome the surface tension, resulting in the formation of larger fibers. This also caused slow solvent evaporation, which led to the formation of fiber junctions and beads (Figure 1e). The Gel concentration was further increased to 30 wt. %; however, the electrospinning liquid viscosity was too high and solvent evaporation was too slow, which led to fiber junctions and flat fibers (Figure 1f). These results indicate that a Gel concentration of 15–20 wt. % for electrospinning fibers is optimal.

### 2.2. Influence of CS Concentration on Fibers

**Figure 2:**
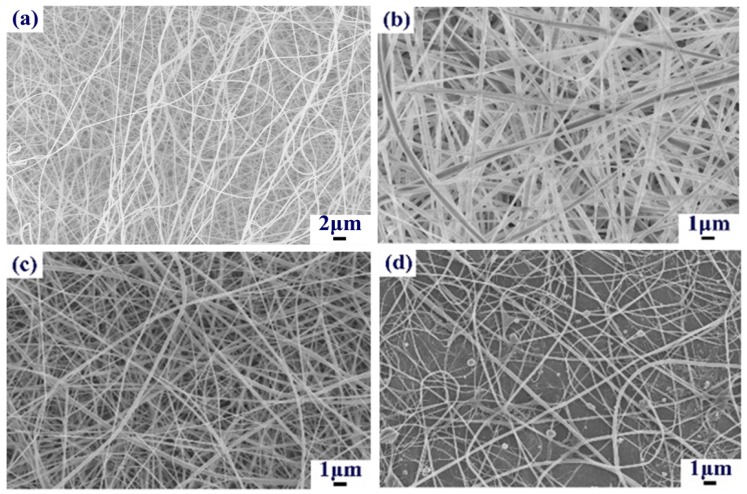
SEM micrographs of fibers prepared with a series of CS concentration; (**a**) 15 wt. % Gel, 0.75 wt. % CS; (**b**) 15 wt. % Gel, 1 wt. % CS; (**c**) 20 wt. % Gel, 0.75 wt. % CS; (**d**) 20 wt. % Gel, 1 wt. % CS.

CS possesses natural antibacterial activity. Figure 2 shows scanning electron microscope (SEM) photographs of fibers after the addition of chitosan for Gel concentrations of 15 wt. % and 20 wt. %. When the Gel concentration was 15 wt. % and the CS concentration was low, electrospinning was easier and electrospun fibers with relatively smooth surfaces were obtained (Figure 2a). As the CS concentration increased, the electrospinning liquid surface tension increased, electrospunfibers exhibited uniformity, and fiber diameters increased (Figure 2b). When the Gel concentration was 20 wt. %, the addition of CS resulted in fine fibers. Junctions were detected in some fibers for low CS concentrations (Figure 2c). As the CS concentration increased, beads were observed during the process of electrospinning (Figure 2d). This may be attributed to the presence of hydrogen bonds in the CS molecules, which increase rigidity and can enhance the mechanical properties of the material. However, increasing the surface tension of the electrospinning liquid also makes electrospinning more difficult, which limits the CS concentration. For a Gel concentration of 15 wt. %, a CS concentration of 1 wt. % was optimal.

### 2.3. Influence of HA Concentration and Particle Size on Fibers

The major inorganic constituent of human bones is HA. Therefore, HA particles were combined with Gel (Gel concentration, 15 wt. %) and CS (CS concentration, 1 wt. %) to enhance fiber biocompatibility. In addition, CS and HA tend to interact with each other, through hydrogen bonds between –NH_2_ and –OH as well as chelation between –NH_2_ and Ca^2+^ [24], to make more uniform fibers. We evaluated HA particles to determine what effect concentration (2 wt. %, 5 wt. %, and 8 wt. %) and particle size (12 μm and 60 nm) has on fiber morphology.

**Figure 3:**
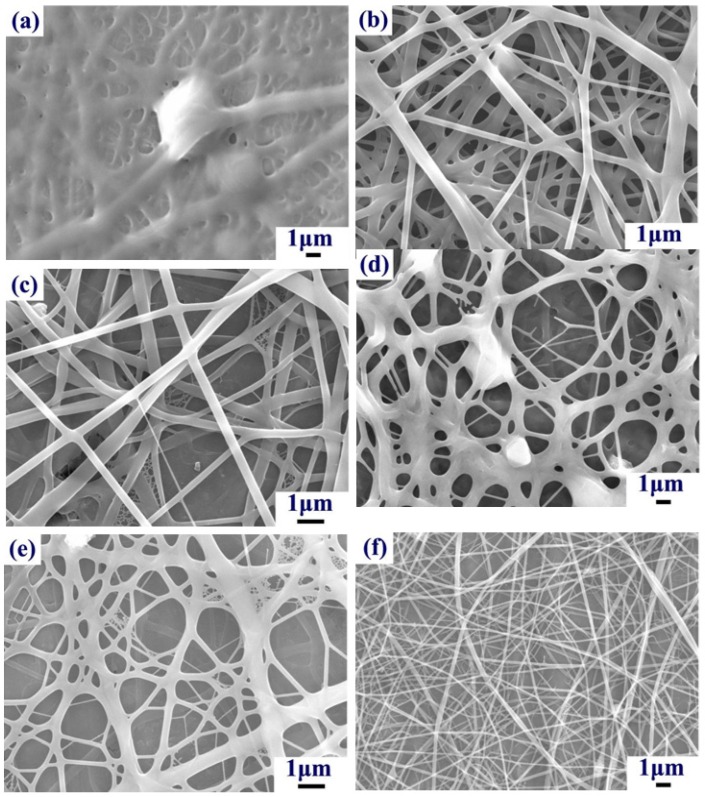
SEM micrographs of fibers prepared with different HA concentration and HA particle size; (**a**) 2 wt. % 12 μm; (**b**) 5 wt. % 12 μm; (**c**) 8 wt. % 12 μm; (**d**) 2 wt. % 60 nm; (**e**) 5 wt. % 60 nm; (**f**) 8 wt. % 60 nm.

**Figure 4:**
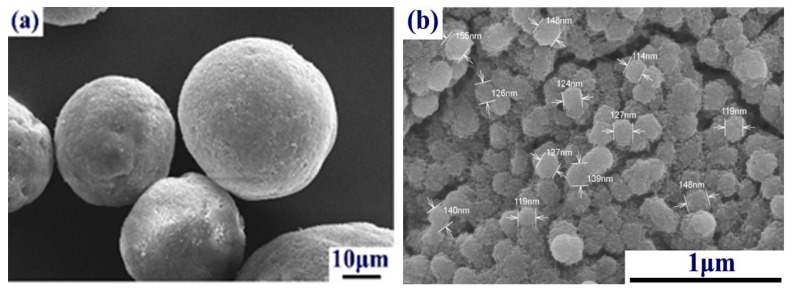
SEM micrographs of HA with a size of 12 μm, before (**a**) and after (**b**) dissolution.

For the HA (12 μm, 2 wt. %) electrospinning solution, the solvent content was relatively high and not completely volatile. This resulted in fiber junctions (Figure 3a). As the HA concentration increased, the number of junctions in the fibers decreased, and gradually more uniform and smooth fibers were produced (Figure 3b,c). HA particles at 12 μm exhibit corrosion in acidic environments, which can lead to HA grain refinement. As shown in Figure 4, grain refinement of HA in acidic conditions was observed for an average particle size of 149 ± 29 nm. Therefore, HA particles can be completely enclosed by the fiber due to the 1 μm fiber diameter.

For HA particles of 60 nm, nanoparticle aggregation resulted in more junctions in the fibers than those observed for HA particles of 12 μm (Figure 3d,e). As the HA concentration was increased to 8 wt. %, the number of junctions decreased (Figure 3f), but the electrospinning solution exhibited HA sedimentation.

Based on these results, the particle size of HA had a significant effect on composite fiber morphology. When HA particles were too small (on the order of nanometers), an aggregation effect was observed. This resulted in a non-uniform electrospinning solution and uneven fibers. When the HA particle size was increased to the micrometer scale, this aggregation effect disappeared. The electrospinning solution was uniform, and the composite fiber morphology improved. In summary, composite fibers can be optimized using a HA particle size of 12 μm and HA concentration of 5 wt. %.

### 2.4. Influence of the GO Concentration on Fibers

**Figure 5:**
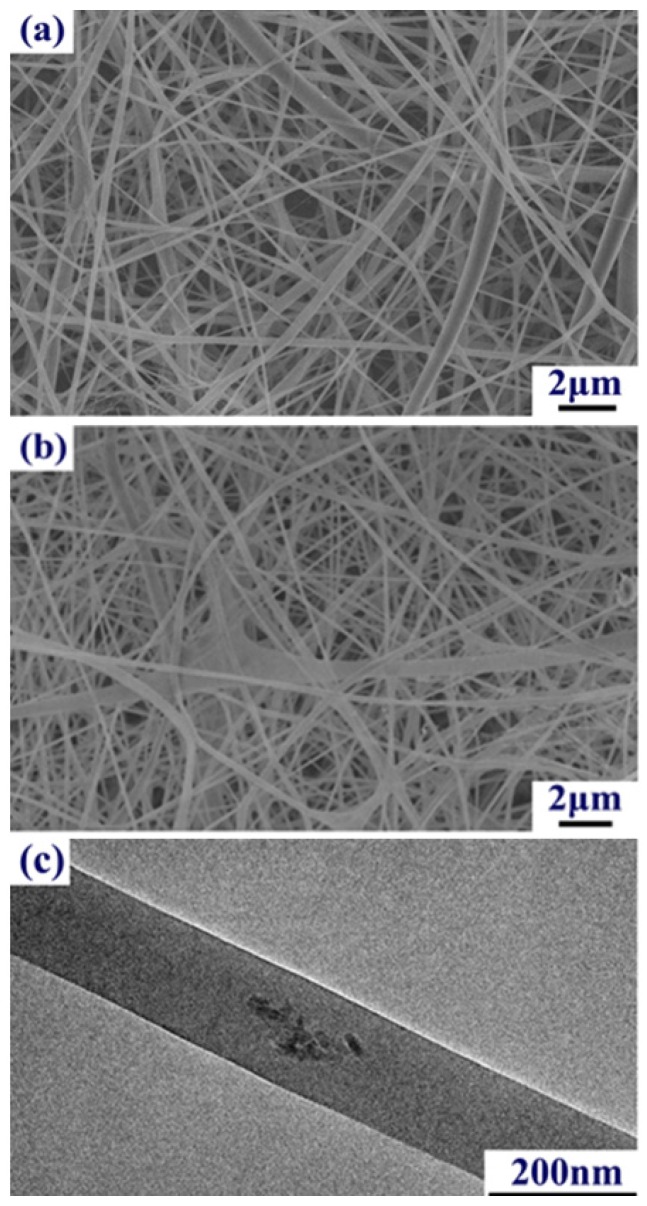
SEM micrographs of fibers with GO and RGO; (**a**) 2 wt. % GO; (**b**) 2 wt. % RGO; and TEM micrographs of the composite fiber (**c**).

The goal of this study was to develop a human bone material with antimicrobial activity. CS possesses weak antimicrobial properties, while GO has excellent antibacterial properties. Accordingly, GO was added to the electrospinning solution with 15 wt. % Gel, 1 wt. % CS, and 5 wt. % HA (12 μm). Figure 5a,b show SEM images of uniform, composite fibers formed from the aforementioned ternary compound electrospinning solution after the addition of 2 wt. % GO and 2 wt. % RGO, respectively. However, if the concentrations of GO and RGO were to be increased in the electrospinning solution, solvent evaporation would cause blockage of the needles during electrospinning. Figure 5 shows that the composite fibers with RGO exhibit bonding (Figure 5b), the GO composite fibers are good (Figure 5a), and the inorganic phase was completely covered in the fiber, as seen by transmission electron microscopy (Figure 5c).

**Figure 6:**
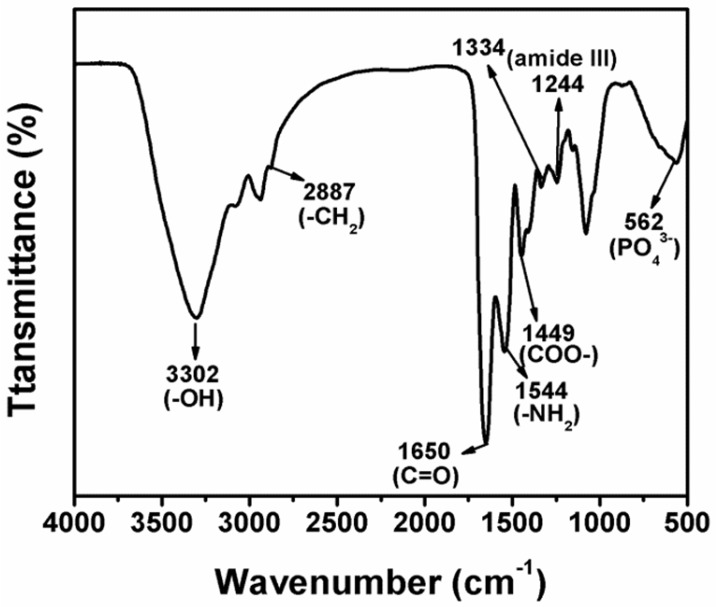
FTIR spectra of Gel/CS/HA/GO composite fibers.

Figure 6 shows the Fourier transform infrared (FTIR) spectrum of Gel/CS/HA/GO composite fibers. The peaks observed at 562 cm^−1^ correspond to the stretching vibration bands of P–O from PO_4_^3−^ in HA. The peaks appearing at 2887, 1650, and 1544 cm^−1^ are attributed to methylene (–CH_2_), C=O in the amide group (amide I band), and the NH-bending vibration in the amide group from chitosan, respectively. The characteristic IR band for amide III is at 1244 cm^−1^, and carboxylate from Gel is at 1449 cm^−1^. The existence of a chemical bond between the carboxylate group in Gel and the Ca^2+^ ion in HA that binds the particulate-reinforced composite together was confirmed by the characteristic IR band at 1334 cm^−1^. FTIR studies show that there was strong interaction between HA, CS, Gel, and GO networks in the composite fibers. Redshifting of the characteristic amine (1660 cm^−1^) and C=O (1592 cm^−1^) bands in chitosan were caused by electrostatic interactions between –NH_3_^+^ and PO_4_^3−^, as well as between C=O– and Ca^2+^ in Gel/CS/HA/GO composite fibers. In summary, the structure of the four species has not changed, which means the composition of the composite fibers has not changed.

### 2.5. Test of Antibacterial Properties

Based on the above experiments, a Gel concentration of 15 wt. %, CS concentration of 1 wt. %, HA (12 μm) concentration of 5 wt. %, and GO concentration of 2 wt. % were optimal conditions. Gel/CA/HA/GO composite fibers were prepared using these concentrations to evaluate antimicrobial properties.

**Figure 7:**
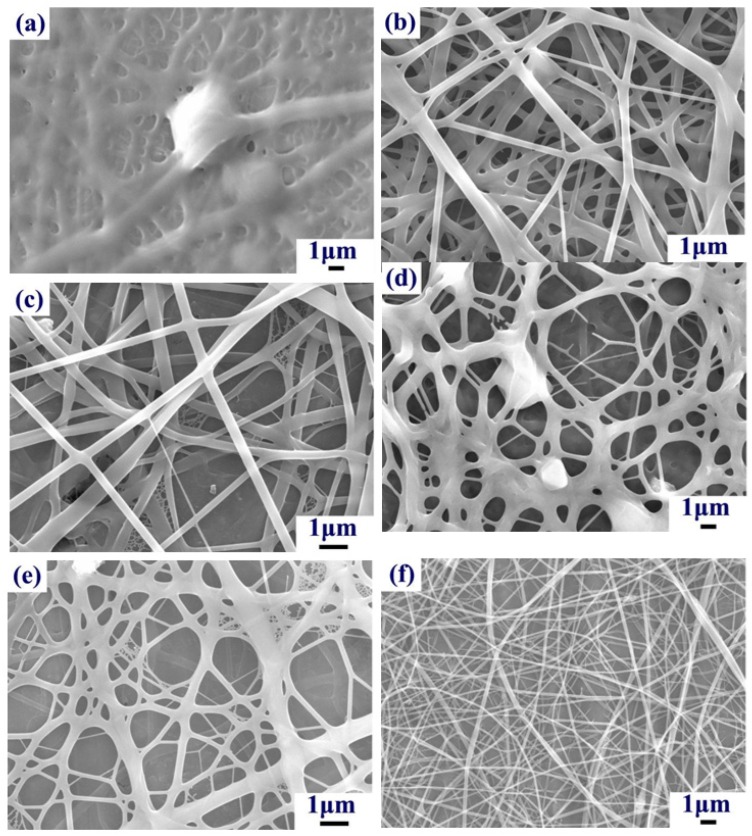
The composite fibers antibacterial test results; (**a**) and (**d**) Gel/CS/HA; (**b**) and (**e**) Gel/CS/HA/RGO; (**c**) and (**f**) Gel/CS/HA/GO; (**a**–**c**) *Escherichia coli* and (**b**–**d**) *Staphylococcus* *albus*.

To investigate the antibacterial effects of the composite fibers against *Escherichia coli* (*E. coli*) and *Staphylococcus* *albus* (*S. aureus*), the spread plate method was used for a qualitative antibacterial analysis. After the bacteria were cultivated for 24 h, the Gel/CS/HA/GO surface did not show *E. coli* colonies (Figure 7c), and Gel/CS/HA/RGO had a few colonies (Figure 7b). The surface of a blank sample of Gel/CS/HA (Figure 7a) had some colonies that were stacked to form a lawn. For antimicrobial properties against *S. aureus*, the Gel/CS/HA/RGO and Gel/CS/HA surfaces had more colonies than the other surfaces, and showed no significant macroscopic differences between samples (Figure 7d,e), while the Gel/CS/HA/GO surface exhibited significantly fewer colonies (Figure 7f). These results indicated that and Gel/CS/HA/GO had stronger antibacterial ability against *E. coli* and *S. aureus*. Meanwhile, the Gel/CS/HA/GO is far more effective against Gram-negative bacteria than against Gram-positive. These results may result from the distinct structure of the cell wall between Gram-positive and Gram-negative bacteria. Gram-positive bacteria contain a thick peptidoglycan layer (20–80 nm) on the outside of the cell wall, and lack an outer membrane. In contrast, the cell wall of Gram-negative bacteria is composed of a thin peptidoglycan layer (7–8 nm) with an additional outer membrane. The thick peptidoglycan of Gram-positive bacteria is a meshlike polymer consisting of amino acids and sugars and may also include other components, such as teichoic and lipoteichoic acids. Therefore, the peptidoglycan layer protects against antibacterial agents, such as antibiotics, toxins, chemicals, and degradative enzymes.

**Figure 8:**
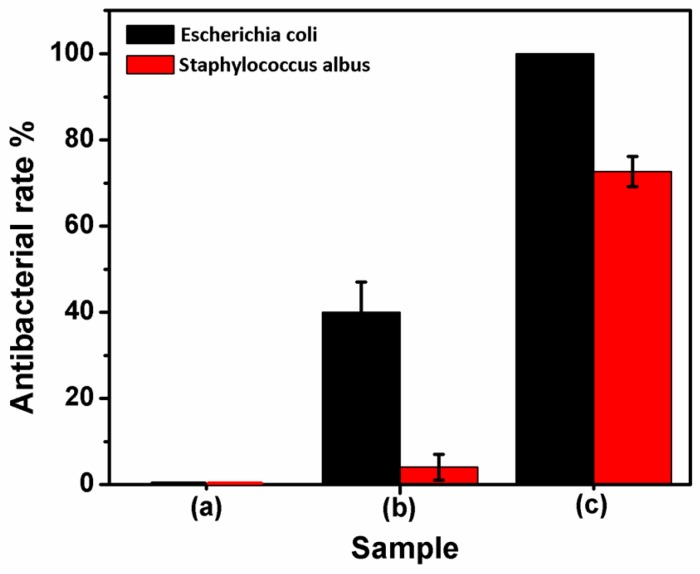
The composite fibers’ antibacterial rate of *Escherichia coli* (black histograms) and *Staphylococcus albus* (red histograms); (**a**) Gel/CS/HA; (**b**) Gel/CS/HA/RGO; and (**c**) Gel/CS/HA/GO.

A quantitative analysis was performed to evaluate the antibacterial properties of the composites, and the results are summarized in Figure 8. For Gel/CS/HA/GO, the *E. coli* antibacterial rate was 100% and for Gel/CS/HA/RGO, the antibacterial rate was only 38.6%. The Gel/CS/HA/GO *S. aureus* antibacterial rate was 73.2%, while Gel/CS/HA/RGO exhibited a weaker antibacterial effect, i.e., 3.4%. The results of the quantitative and qualitative analyses demonstrated that Gel/CS/HA/GO has strong antibacterial properties against *E. coli*, and has good antibacterial properties against *S. aureus*.

### 2.6. Adsorption Performance of BSA

After the addition of GO, the composite fiber morphology was more uniform and excellent antibacterial activity was observed. Protein adsorption has a substantial effect on osteoblasts. The effects of GO on the BSA adsorption ability of the composite fibers were examined. Specifically, the effects of pH and initial concentration of the BSA solution on the protein adsorption of the composite fiber were examined.

**Figure 9:**
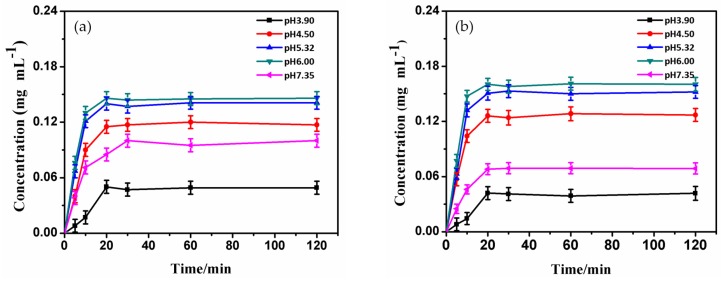
Protein adsorption curve of composite fibers immersed in the BSA solution with different pH values: (**a**) Gel/CS/HA; and (**b**) Gel/CS/HA/GO.

The pH of the BSA solution had a significant effect on composite fiber protein adsorption. For various pH values, variation in the BSA concentration versus time is shown in Figure 9a,b. For Gel/CS/HA and Gel/CS/HA/GO, the adsorption capacity all increased sharply during the first 20 min, then levelled-off gently, and finally reached equilibrium. The adsorption ability of the composite fiber increased as the solution pH increased. When a pH value of 3.90 was applied, protein adsorption on the surface of all composite fibers was minimal. Adsorbed protein for composite fibers was maximal for a pH value of 5.32–6.00. From the two figures we can see that the Gel/CS/HA/GO composite balance concentration is greater than that of Gel/CS/HA. Thus, we mainly investigated the effect of initial BSA concentration on the Gel/CS/HA/GO composite fibers’ protein adsorption.

**Figure 10:**
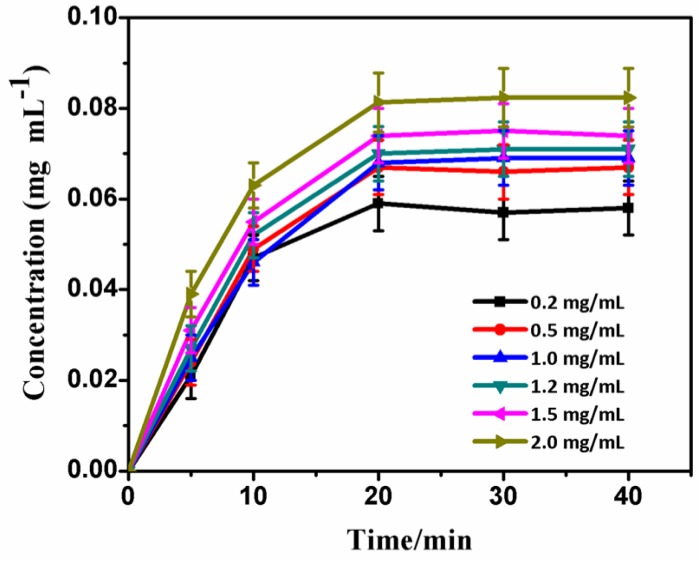
Protein adsorption curve of the Gel/CS/HA/GO composite fibers immersed in the BSA solution with different concentrations.

The initial BSA concentration also had a significant effect on composite fiber protein adsorption. Variation in BSA concentration over time was observed when the composite fibers were immersed in BSA solutions with a pH value of 7.35. The results are summarized in Figure 10. The saturated protein adsorption on the surface of the composite fiber increased as the initial BSA concentration increased. When the initial concentration was constant, the surface of the composite reached the adsorption equilibrium in 20 min.

## 3. Discussion

Gel/CS/HA/GO composite nanofibers were prepared using electrospinning to evaluate their antibacterial properties. Recent studies have emphasized [25] the strong influence of substance concentration in electrospinning liquids on fiber morphology. Therefore, the effects of the composition on fiber morphology and antibacterial properties were investigated.

According to a recent report [26,27], the conductivity of the electrospinning liquid is mainly determined by the ionized salt type, polymer type, and concentration. Some ionized substances added to the electrospinning solution do not change the electrically-neutral property of the electrospinning solution. However, decomposition into positive and negative ions can obviously change the electric charge density of the electrospinning solution, improve electrical conductivity, and affect the morphology and diameter of fibers. In our study, as the HA concentration increased, the dissolution of HA increased in an acidic solution, and the inorganic ion concentration increased in the electrospinning solution, thereby enhancing the conductivity of the electrospinning solution, resulting in thinner fibers. Essentially, natural polymers, including polyelectrolytes, like –NH_2_, generates –NH_3_^+^ when CS is added to an acidic solution. This increases the electrospinning solution conductivity. A hybrid electrospinning solution consisting of HA and CS, N and Ca^2+^ can be hybridized by the protonation of CS molecules [24], resulting in complex formation, but this does not change the charge density of the electrospinning solution. Therefore, the reaction between CS and the inorganic ions does not influence spinnability. However, various CS and inorganic ion concentrations affect the conductivity of the electrospinning solution and, thus, influence the morphology and diameter of electrospun fibers. Gel shows a positively-charged point below isoelectric, which is similar to CS because the isoelectric point of Gel is between 6.00 and 8.00. When Gel is added to an acidic solution it is positively charged, which increases the electrospinning solution conductivity. A hybrid electrospinning solution that consists of HA and Gel, N and Ca^2+^ can be hybridized in the protonation of Gel molecules, resulting in complex formation.

In this experiment, for a lower CS content in composite fibers, the antimicrobial properties were mainly attributed to GO and RGO. According to recent reports, both GO and RGO have shown good antimicrobial properties, and the antibacterial mechanism is a result of oxidative stress and cell membrane damage. Oxidative stress in target cells is caused by the generation of reactive oxygen species. Antioxidant enzymes in the cell can be used to reduce and eliminate reactive oxygen species. If homeostasis is not achieved, cellular macromolecules, such as proteins, DNA, and lipids, can be damaged [28]. Cell membrane damage via physical interactions with sharp-edged graphene is another possible antibacterial mechanism [29]. Feng et al. [30] found that *E. coli* can interact directly with GO to induce the loss of bacterial membrane integrity and glutathione oxidation, suggesting that GO antimicrobial action contributes to both membrane disruption and oxidative stress. Additionally, the antibacterial mechanism of grapheme-based materials largely depends on the surface of nanomaterials; when GR is well dispersed in the composite material, its antibacterial effect is stronger. In this experiment, GO had good hydrophilicity, which promotes dispersion in a composite fiber. Accordingly, evenly dispersed GO has shown strong antibacterial activity [31].

Additionally, the effects of different pH values on the properties of protein adsorption have been investigated. When a pH value of 3.90 was applied, protein adsorption on the surface of composite fibers was minimal. Since the pH was lower than the BSA isoelectric point (4.90), BSA had a positive charge, and the gelatin isoelectric point was 6.00–8.00. In other words, when the pH value ≤4.90, gelatin also has a positive charge, and electrostatic repulsion interactions between the surface of composite fibers and BSA decreases the adsorption capacity of the surface of the composite fibers. When the pH exceeds the BSA isoelectric point (4.90), BSA has a negative charge. When 4.90 ≤ pH ≤ 6.00, gelatin has a positive charge, and the electrostatic attraction between BSA and Gel is beneficial for protein adsorption; accordingly, adsorbed protein for composite fibers was maximal for a pH value of 5.32–6.00. pH values in the range of 5.32–7.35 characterize the normal physiological environment of the human body. In an alkaline environment, BSA and Gel are negatively charged and repel each other, which is not conducive to protein adsorption. Meanwhile, when investigating the effect of initial concentration on protein adsorption, the results demonstrated that Gel/CS/HA/GO composite fibers have good protein adsorption performance. Furthermore, these scaffolds and their effect in supporting stem cells for bone regeneration will be reported in the future.

## 4. Experimental Section

### 4.1. Materials

Acetic acid (CH_3_COOH) and 30% hydrogen peroxide (H_2_O_2_) were produced by Tianjin Yong Sheng Chemical Co., Ltd. (Tianjin, China). CS (low molecular weight) with a degree of deacetylation of about 91% was obtained from Sigma (St. Louis, MO, USA). Gel and sulfuric acid (H_2_SO_4_) were supplied by Beijing Chemical Factory (Beijing, China). HA, with average particle sizes of 12 μm and 60 nm, was obtained from Shanghai Blue Reagent, Co., Ltd. (Shanghai, China). Graphite was obtained from Shanghai Mountain Pu Chemical Co., Ltd. (Shanghai, China). Potassium permanganate (KMnO_4_) was obtained from Xian Bo Station Always Sells On Commission (Shanxi, China). Hydrazine hydrate (N_2_H_4_·H_2_O) was produced by Luoyang Chemical Reagents (Luoyang, China). Bovine serum albumin (BSA) was supplied by Shanghai Blue Technology Development Co., Ltd. (Shanghai, China).

The following instruments were used in the study: a TL01 Electrostatic Spinning Machine (Shenzhen Tong Li Wei Technology Co., Ltd., Shenzhen, China), a scanning electron microscope (SEM, Carl Zeiss, LEO-1430 vp; Oberkochen, Germany), a transmission electron microscope (TEM, JEOL JEM-2100-f; Tokyo, Japan), a Fourier transform infrared spectrometer (FTIR, BRUKER VERTEX70; Brook, Germany), and an ultraviolet spectrophotometer (U3310; Hitachi, Tokyo, Japan).

### 4.2. Preparation of Graphene Oxide and Reduction of Graphene Oxide Using the Hummers Method

Graphite (5.0 g) was added to a 500 mL distillation bottle with concentrated H_2_SO_4_ (115 mL). KMnO_4_ (25 g) was added to the distillation bottle very slowly with vigorous stirring for 2 h at 0 °C. The mixture was then transferred to an oil bath with lateral flow agitation at 35 °C overnight. Water was then added slowly to an oil bath at 90 °C, followed by heating for 30 min (the solution color immediately turned from black to chocolate brown). At this time, H_2_O_2_ (15 mL) was added. The solution was stirred for 30 min, filtered, and washed several times with deionized water and hydrochloric acid (HCl) solution until the pH was nearly neutral, and then dried overnight using a drying oven at 60 °C to obtain GO.

Aqueous GO was added to a flask that was connected with condenser pipe backflow devices. Hydrazine hydrate (2 mL) was added. Agitation backflow was performed for 24 h at 100 °C in oil bath conditions. The product was washed with ethanol and distilled water three times; it was then dried to a constant weight in a vacuum at 60 °C to obtain RGO.

### 4.3. Configuration of Electrospinning Solution

CS (1 wt. %) was added to 20% (*v*/*v*) acetic acid (20 mL) and stirred for 12 h. Gelatin (15 wt. %) was then added and dissolved at 60 °C in a water bath. HA particles (5 wt. %), powdery GO (2 wt. %), or RGO (2 wt. %) were added, the temperature was maintained, and the solutions were stirred for 30 min, resulting in the uniform dispersion of HA and GO in the solution. Configurations with particular concentrations of electrospinning liquid were obtained.

### 4.4. Preparation of Electrospun Fibers

A dry plastic syringe was filled with the solution and connected to a blunt-end stainless steel needle (#6). The syringe was fixed to a syringe pump. A stainless steel plate covered with aluminum foil (20 × 13 cm) was used as a collector and grounded. The fixed distance from the syringe needle tip to the aluminum foil was 15 cm. The syringe pump rate was adjusted, and a uniform flow rate of 2 mL·h^−1^ was used. The high voltage power supply was opened and the electrospinning voltage was adjusted to prepare electrospinning fibers. The laboratory temperature was 25 °C and the relative humidity was 45%–55%. The electrospunfibers were dried for three days under a vacuum at room temperature to remove residual solvents.

### 4.5. Test of Antibacterial Properties

A Gram-negative species (*Escherichia coli*) and a Gram-positive species (*Staphylococcus aureus*) were used to examine antibacterial properties. Qualitative and quantitative evaluations of the antibacterial activity of the Gel/GO/CS/HA composite fibers were performed. The spread plate method was used for qualitative analysis and the film adhering method was used for quantitative analysis. The antibacterial experiment included three groups: Gel/CS/HA, Gel/CS/HA/RGO, and Gel/CS/HA/GO. All experiments were repeated at least three times.

For the qualitative analysis, *E. coli* and *S. aureus* were plated on LB (Luria-Bertani) culture medium. The bacteria were aerobically cultivated at 37 °C for 24 h. Adequate amounts of bacteria were applied using inoculation loops, and were incubated in liquid medium for 24 h to obtain bacterial solutions. Secondly, the two bacteria were adjusted to a concentration of 1 × 10^8^ cell mL^−1^ (10 mL) using phosphate-buffered saline (PBS), and the cultures were incubated at 37 °C with shaking at 200 rpm for 12 h. After shaking well, the 100 μL coated tablet was removed and incubated for 24 h at 37 °C in a constant temperature incubator. Plates were examined for bacterial growth and images were obtained.

For the quantitative analysis, the antimicrobial rate of samples was evaluated using the sample surface adhesion method for the two types of bacteria plated on the LB culture medium. The bacteria were cultivated at 37 °C for 24 h. This process was repeated three times to obtain pure colonies. One bacterium was inoculated in liquid medium at 37 °C with shaking at 220 rpm for 12 h. The two bacteria were configured to 3.0 × 10^7^ cell mL^−1^ using PBS and diluted 10^3^ times. The measured sample was loaded onto a Petri dish and sterile water was added to the bottom of the dish to prevent evaporation. Next, 50 μL drops were added to the surface and cultured for 12 h at 37 °C. The bacterial fluid was added to PBS (500 μL), mixed well, and 50 μL was plated. After cultivation for 12 h, colony-forming units were obtained. The antibacterial activity of the samples was estimated by calculating the antibacterial rate of samples based on the following formula. Gel/CS/HA was used as the control group, and three parallel experiments were conducted in order to obtain the average rate.

Antibacterial rate = (colonies of control group − colonies of experimental group) × 100%/colonies of control group

### 4.6. Protein Adsorption

BSA was prepared with phosphate buffer solution (PBS, pH 7.40), and a 10 times concentration of PBS solution formula as shown in Table 1. The samples with dimensions of 1 cm × 1 cm were incubated in 10 mL of the BSA solution in a centrifuge tube at room temperature. The change in the concentrations of BSA in the solution was determined by measuring the absorbance at 278 nm using an ultraviolet spectrophotometer.

### 4.7. Statistical Analyses

All data are presented as means ± standard deviation. Statistical analysis was carried out using a one-sample *t*-test (assuming unequal variance). The difference between two sets of data was considered statistically significant when *p* < 0.05.

## 5. Conclusions

In summary, we demonstrated that quaternary Gel/CS/HA/GO composite fibers could be prepared by a simple electrospinning method. The powdery HA and GO were uniformly dispersed into Gel and CS matrix. Furthermore, the Gel/CS/HA/GO composite fibers displayed better antibacterial effects against *E. coli* and *S. aureus* compared with Gel/CS/HA composite fibers. This study observed that the composite fibers have a good adsorption capacity of BSA at the normal physiological environment of the human body. The protein adsorption on the surface of the composite fiber increased as the initial BSA concentration increased. At a certain initial concentration, the surface of the composite can reach the adsorption equilibrium, namely, the maximum adsorption value. These scaffolds and their effect in supporting stem cells for bone regeneration will be reported in the future.

## Figures and Tables

**Table 1:** Ten times concentration of phosphate buffer solution.

Reagent	NaCl	KCl	KH_2_PO_4_	Na_2_HPO_4_
Concentration (g·L^−1^)	80.0	2.0	2.4	14.4
